# Depression and Anxiety Symptoms of British Adoptive Parents: A Prospective Four-Wave Longitudinal Study

**DOI:** 10.3390/ijerph16245153

**Published:** 2019-12-17

**Authors:** Rebecca E. Anthony, Amy L. Paine, Katherine H. Shelton

**Affiliations:** 1 Centre for the Development and Evaluation of Complex Interventions for Public Health Improvement (DECIPHer), School of Social Sciences, Cardiff University, 1-3 Museum Place, Cardiff CF10 3BD, UK; AnthonyRE@cardiff.ac.uk; 2 School of Psychology, Cardiff University, Tower Building, 70 Park Place, Cardiff CF10 3BD, UK; paineal@cardiff.ac.uk

**Keywords:** adoption, parent mental health, parent competency, child psychopathology

## Abstract

The mental health of birth parents has gained attention due to the serious negative consequences for personal, family, and child outcomes, but depression and anxiety in adoptive parents remains under-recognized. Using a prospective, longitudinal design, we investigated anxiety and depression symptoms in 96 British adoptive parents over four time points in the first four years of an adoptive placement. Depression and anxiety symptom scores were relatively stable across time. Growth curve analysis showed that higher child internalizing scores and lower parental sense of competency at five months post-placement were associated with higher initial levels of parental depressive symptoms. Lower parental sense of competency was also associated with higher initial levels of parental anxiety symptoms. Parents of older children and those with higher levels of parental anxiety and sense of competency at five months post-placement had a steeper decrease in depressive symptoms over time. Support for adoptive families primarily focuses on child adjustment. Our findings suggest that professional awareness of parental mental health post-placement may be necessary, and interventions aimed at improving parents’ sense of competency may be beneficial.

## 1. Introduction

Parenthood, though often joyful, is a major life transition marked by a range of stressors that can result in increased anxiety and low mood [1]. Parents’ symptoms of depression and anxiety are associated with serious negative consequences for personal, family, and child developmental outcomes in both genetic [2] and non-genetically related families [3]. Although there are hundreds of studies on birth parents’ adjustment to parenthood, comparatively few have examined the mental health of adoptive parents [4,5]. Given that parental depression and anxiety have serious implications for children’s outcomes beyond infancy [6], and depression in adoptive parents is associated with placements in the UK breaking down [7], there is a vital need for better understanding of adoptive parents’ mental health. Most studies of adoptive parent wellbeing have been conducted in the US using maternal cross-sectional data; where longitudinal data is available, it is generally short-term and conducted with samples of private and/or international adoptions [5]. Therefore, to effectively inform the development of policies and practice in the UK, we investigated trajectories and predictors of adoptive parents’ symptoms of depression and anxiety over the first four years following the adoption of a child from the public care system.

Rates of adoptive parent depression are estimated to be as high as 32%, but this estimate varies greatly across studies and contexts [5,8,9,10,11,12,13]. Although adoptive mothers do not experience many of the risk factors for depression in biological mothers, such as hormonal changes or delivery complications [14], adoptive parents experience the same challenges that any new parent faces, including increased levels of stress, lack of sleep, and alterations in their intimate partner relationships [9]. Parents who adopt children from the UK public care system may face unique challenges; for example, in the US, most children are adopted by their foster carers (56%), and stranger/matched adoptions are less common (14%) [15], yet the reverse is true in the UK; most parents adopt their child as ‘strangers’ (85%) [7,16]. As such, adoptive parents contend with establishing an emotional relationship with their child, who may not have had a stable, positive experience of family life [17] and may also struggle to understand their new family circumstances [18]. In the UK, communicative openness—open and honest communication that embraces the meaning of adoption—is encouraged via the provision of life story books in line with statutory guidance [19]. Although communicative openness has been associated with child wellbeing [20], some parents find discussing the subject of adoption and their child’s birth parents challenging [7].

Not only do adoptive parents have to adapt to the psychological and practical needs of their child, but they may have faced a considerable number of challenges and specific pressures in creating their family through adoption. Prior to the adoption, parents may have experienced a protracted period of diagnostic and treatment procedures related to infertility and may continue to grieve the loss of a hoped-for birth child in the post-adoptive period [21]. Adoptive parents undergo agency evaluations of parental fitness and, if approved, must wait an indefinite period to be matched [22]; a process that, in the UK, can take around two years to complete [23]. They may also encounter delays associated with court applications and hearings, and prospective parents face the possibility that their application may be contested, and the child may not be relinquished by birth parents [24].

To our knowledge, the first study to examine adoptive parent depression over a substantial amount of time was carried out in the US in 2014; parents who adopted children from foster care maintained low, non-clinical levels of depressive symptoms and parenting stress over time [25]. Another US study with mostly private adoptions [10] found that the percentage of parents who were clinically depressed was highest immediately after placement of the child (from 9.5% pre-placement increasing to 11.3% when the placement occurred). Subgroups of parents were characterized according to the trajectories of their depressive symptoms. Most adoptive parents (71%) maintained low levels of depressive symptoms over time; however, two subgroups of parents were above the threshold for depressive symptoms at placement, and three subgroups of parents were above the threshold at six months post-placement.

Few studies have examined symptoms of anxiety in adoptive parents. In some exceptions, evidence shows adoptive mothers experience fewer symptoms of social anxiety, panic, and traumatic intrusions, and greater well-being compared to birth mothers [12], and anxiety symptom scores are lower than people in the general population [7]. In one study of US domestic adoptions, levels of anxiety symptoms in adoptive parents declined over time [26]. However, to our knowledge, no studies have explored adoptive parents’ symptoms of anxiety and depression over more than three time points. Given that adoptive parents’ mental health problems may rise and fall following the placement of their child(ren) [10], we investigated adoptive parents’ symptoms of depression and anxiety at four time points over the first four years of a placement to explore the possibility of non-linear time patterns in adoptive parents’ mental health problems.

### 1.1. Risk Factors for Depression and Anxiety in Adoptive Parents

Most children adopted from the public care system will have experienced abuse and/or neglect within their birth family [7,27], and this may be compounded by experiences whilst in the public care system, such as experiences of repeated moves between foster carers. Children who are adopted when they are older are more likely to have experienced abuse and/or neglect and multiple placements associated with behavioral and emotional problems [28]. Both foster and adoptive parents consistently rank children’s behavior problems as the most difficult challenge [29], and unsurprisingly, the severity of emotional and behavioral issues among children are associated with higher levels of parental depressive symptoms and parenting stress [30,31,32]. Many adoptive parents describe feeling ill-equipped to manage their children’s behavior [33]. Furthermore, although there is evidence to suggest that children tend to fare at least as well, or better when placed with their siblings [34], caring for sibling groups may have implications for the parenting task [35].

Child characteristics can impact a parent’s sense of competency: a parent’s perception of their skills (or, self-efficacy), the pleasure or motivation derived from parenting, and their satisfaction with the role [36]. Mothers who perceive their children as more difficult (i.e., higher levels of child non-compliance) exhibit lower feelings of parenting efficacy than mothers who do not [37]. In a longitudinal study examining trajectories of child negative emotionality, parenting efficacy, and over-reactive parenting among adoptive families [38], child negative emotionality was associated with decreases in maternal efficacy and parent over-reactivity. Very few studies focus on the role of sense of competency in adoptive parents; however, Foli’s mid-range theory of post-adoptive depression [9,39] asserts that adoptive parents may set unfulfilled/unrealistic expectations of themselves and family life (e.g., of their parenting competence; the bond they will have with their child; the support they will receive from family and friends), and that dissonance between expectations and reality can lead to depression.

### 1.2. The Current Study

In the present study, we addressed the need for longitudinal investigation of parents’ post-adoptive symptoms of depression and anxiety. We applied multilevel growth models to study symptoms of anxiety and depression of parents who adopted children from the UK public care system at four intervals over a four-year period after adoption. The aims were threefold: (1) to profile depression and anxiety symptoms in adoptive parents; (2) to plot trajectories of depression and anxiety symptoms in adoptive parents during the first four years of an adoptive placement using unconditional growth models; and (3) to investigate factors associated with initial levels of adoptive parents’ symptoms of anxiety and depression and the rise and/or fall of symptoms over time using conditional growth models. We expected that parents who adopted a child who was older at the time of placement and had more emotional and behavioral problems would have more symptoms of depression and anxiety and that parents’ sense of competency would be associated with fewer symptoms. Considering the inconsistency of evidence regarding adopting a sibling group, we made no predictions about the direction of effects.

## 2. Method

### 2.1. Design

We used data from the Wales Adoption Cohort Study, a prospective longitudinal study of a national sample of children placed for adoption from care in Wales between 1 July 2014 and 31 July 2015. Of the 118 adoptive families eligible for study inclusion and who contacted the research team, 96 returned the initial questionnaire at 5 months post-placement (81% response rate). These 96 families formed the study panel, and were followed up longitudinally at 4 time points post-placement. Baseline data concerning child characteristics, their pre-adoption experiences and support needs were obtained by reviewing child adoption reports (CARs). The present study used the questionnaire follow ups that took place at approximately 5-, 21-, 36-, and 48-months post-placement (Waves 1 to 4 [W1 to 4]), respectively). Ethical permission for the study was granted by the School of Social Sciences Research Ethics Committee at Cardiff University and permission to access the CARs was obtained from the Welsh Government (see [24] for more details).

### 2.2. Procedure

#### 2.2.1. Questionnaire Data

At each time point, families completed a questionnaire concerning sociodemographic information, pre- and post-adoption experiences, the child and parent’s mental health, and adoptive family relationships. Where groups of siblings were placed together, parents were asked to report on the oldest child in the placement. Of the 96 families that participated in the study at W1, 81 (84.4%) participated in the second wave, 73 (76.0%) participated at W3, and 68 (70.8%) participated at W4. Questionnaires were completed by either an adoptive mother (87.5% at W1, 87.7% at W2, 97.3% at W3, 92.6% at W4) or father.

#### 2.2.2. Case File Data

Information pertaining to the pre-adoptive history of the child was gathered from their child adoption report (CAR). CARs are completed by social workers, who record information based on their work with birth parents, contact with foster carers, liaison with other professionals (e.g., police, health visitors, and medical officers), and reviews of historical social services records. Researchers worked on-site at the local authority offices and gathered information from electronic and hard-copy formats of CAR records from the period of study. Adoption reports for all children placed for adoption between 1 July 2014 and 31 July 2015 were reviewed (*N* = 374).

### 2.3. Participants

The adoptive parents had a mean age of 40.67 (*SD* = 6.99, range 22 to 62) years at the time of adoption, and the majority (99%, *n* = 94) were white British. Most parents were in a heterosexual relationship (82%, *n* = 79), 5% (*n* = 5) were in a same-sex relationship, and 13% (*n* = 12) were single adopters. Fifty-seven (59.4%) were first-time parents. At the W1 assessment, there was a median of 4 people living in the home with the child, and most informants were in either full-time or part-time paid work (*n* = 72, 54.2%). The gross family income and education levels were substantially higher than the UK average [40], where 12% earned more than £75,000 per year, and 37% had postgraduate degrees.

Of the children who were reported on by their parents in the longitudinal follow-up questionnaires (*N* = 96), 47 (49%) were female, and were placed for adoption at a mean age of 2.36 (*SD* = 2.20, range 0 to 9 years); 41.2% were removed at birth. Children spent a mean of 522.92 (*SD* = 611.75, range 0 to 2344) days with their birth parents and a mean of 537.09 (*SD* = 285.74, range 203 to 1401) days in care. Four children (4%) were fostered by their adoptive parent(s). Twenty-nine children (30%) were adopted as part of a sibling group. Characteristics of the adopted children in the present study were compared to those of all children placed for adoption in the same time window (*N* = 374). There were no differences found in children’s gender, age, and past experiences of abuse/neglect (all *p*s > 0.05). Attrition analyses showed no differences in sociodemographic characteristics (child gender and age, parent relationship status, education, and income) between those who participated in W1 and 4 of the study (all *p*s > 0.05). Parents of children with higher externalizing problems were less likely to remain in the study at W4 (rated using the Strengths and Difficulties Questionnaire (SDQ) [41]; *p* < 0.01, all other *p*s > 0.05).

### 2.4. Measures

#### 2.4.1. Parent Symptoms of Depression and Anxiety

The Hospital Anxiety and Depression Scale (HADS) is a brief 14-item self-report measure of anxiety and depression [42]. The scale comprises 14 items, seven of which assess anxiety (e.g., “I can sit at ease and feel relaxed”) and seven that measure depression (e.g., “I still enjoy the things I used to enjoy”). The items are scored on a 4-point scale ranging from 0 to 3, with higher scores indicating higher anxiety and depression (maximum score is 21 on each scale). The HADS has good discriminant validity, internal consistency, and concurrent validity [43]. In the present study the HADS was completed at all four time points and had good levels of internal consistency (depression; *α* = 0.741 to 0.798 and anxiety; *α* = 0.808 to 0.860).

#### 2.4.2. Parent Sense of Competency

The Parenting Sense of Competence Scale (PSOC) was used to assess parent sense of competency [44]. This is a 17-item scale with a two-factor structure: satisfaction, which assesses the degree to which an individual enjoys his or her role as a parent; and efficacy, which assesses an individual’s perceived competence in his or her role as a parent, for example, “I meet my own personal expectation for expertise in caring for my child,” and are measured on a 6-point Likert-type scale ranging from strongly disagree to strongly agree. Higher scores represent greater parenting competence, with a total scale score range between 17 and 102. This measure has good reliability [36] and validity [45]. Cronbach’s alpha showed good levels of internal consistency in this study (*α* = 0.83 to 0.86 across 4 time points).

#### 2.4.3. Age Placed for Adoption

The age of children when placed for adoption (in years) was extracted from the case file data, calculated by subtracting the child’s birth date from the date they were placed for adoption.

#### 2.4.4. Child Internalizing Symptoms and Externalizing Problems

Adoptive parents completed the SDQ [41]. We used the internalizing symptoms (sum of emotional and peer problem scales) and externalizing behavior problems (sum of conduct and hyperactivity scales), where higher scores are indicative of more problems (scoring a maximum of 20). The internalizing and externalizing scales had acceptable to good levels of internal consistency across all time points (*α*s ranged from 0.60 to 0.84).

### 2.5. Data Analysis

We first present descriptive statistics and correlations among variables of interest to profile depression and anxiety symptoms in adoptive parents. We then used growth curve analysis to investigate longitudinal trajectories of depression and anxiety symptom scores; growth trajectories were modelled using MPlus version 8 [46]. Growth curve modelling is a broad term referring to a wide array of statistical models for repeated measures data. Growth curve models allow for the estimation of inter-individual variability in intra-individual patterns of change over time [47]. Specifically, growth curve models allow for the estimation of within-subject trajectories of change (growth curve) for a variable, described by two parameters: an intercept (initial level of the variable) and a slope (rate of change over time). Both analytical and simulation results show that growth models are typically characterized by higher levels of statistical power than comparable traditional methods applied to the same data [48]. We show the plotted trajectories of depression and anxiety symptoms in adoptive parents during the first four years of an adoptive placement using sample means and estimated means for the unconditional growth model (e.g., the mean of the trajectory pooling all the individuals within the sample) and conditional growth model (accounting for predictors of interest). In the conditional growth models, we investigated factors associated with initial levels of adoptive parents’ symptoms of anxiety and depression and the rise and/or fall of symptoms over time.

#### 2.5.1. Missingness

The method for handling attrition in the outcome measure depends on whether attrition is considered missing at random (MAR) or missing not at random (MNAR). To assess whether attrition was MAR, a Diggle-Kenward selection model [49] was estimated for the growth processes for parental depression and anxiety. Future dropout was related to the previous outcomes; therefore, the assumption of MAR was not supported. Due to this, data analysis employed selection models for both MAR analysis using maximum likelihood with robust standard errors (MLR) and MNAR using the Diggle-Kenward selection as recommended [50]. Studies have found that the Diggle-Kenward performs better than the MAR based maximum likelihood estimator method under the MNAR mechanism, especially with smaller sample sizes and samples with more than 10% attrition [51]. As the sensitivity analysis showed minimal difference between the MAR and MNAR analyses, only the MAR are reported, and MNAR analyses are available upon request. Missingness was handled in MPlus using full-information maximum-likelihood (FIML) estimation, which uses all the available information for each participant rather than deleting participants or imputing values [52].

#### 2.5.2. Model Fit

Goodness-of-fit statistics were used to evaluate model fit. Acceptable model fit is indicated by a χ^2^/df ratio below or around 3 [53]. Comparative fit (CFI) and Tucker Lewis indices (TLI) close to or above 0.95 [54], a root mean square error of approximation (RMSEA) value below 0.05 [55], and lower values for the Akaike information criterion (AIC) and Bayesian information criterion (BIC) [56]. In the MNAR analyses of growth curve models using the Diggle-Kenward method, CFI, TLI, and RMSEA were not available; therefore, the fit was assessed using the AIC and BIC. Once the best fitting models were identified, we tested associations between variables of interest and the intercept and slope growth terms.

## 3. Results

**Figure 1:**
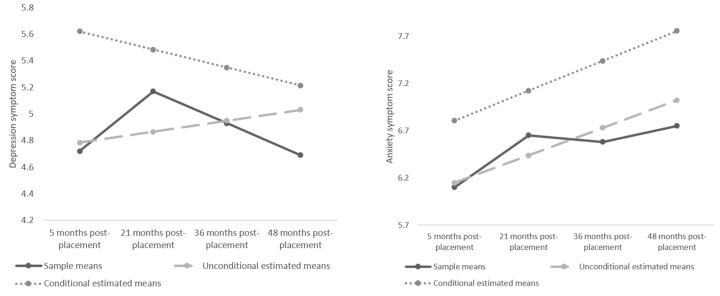
Depression (**left**) and anxiety (**right**) symptoms sample means, estimated unconditional model means and conditional model means.

The mean HADS scores for depression were 4.72 (*SD* = 3.43) at W1 (5 months post-placement), 5.17 (*SD* = 6.65) at W2 (21 months post-placement), 4.93 (*SD* = 6.58) at W3 (36 months post-placement), and 4.69 (*SD* = 3.08) at W4 (48 months post-placement) (see Figure 1). The percentage of parents who met cut-off criteria for probable disorder were 7% (*n* = 7) at W1, 12% (*n* = 9) at W2, 7% (*n* = 5) at W3, and 4% (*n* = 3) at W4. For HADS symptoms of anxiety, the mean scores were 6.10 (3.48) at W1, 6.65 (4.03) at W2, 6.58 (3.63) at W3, and 6.75 (4.20) at W4. The percentage of parents who met cut-off criteria for probable disorder were 10% (*n* = 10) at W1, 17% (*n* = 13) at W2, 16% (*n* = 11) at W3, and 16% (*n* = 11) at W4.

There were no associations between parent demographic characteristics (gender, age, relationship status, and education), child gender, and depression and anxiety symptom scores at any time point. Therefore, due to the sample size restrictions, these were not included as covariates [57]. Descriptive statistics and correlations between variables of interest are summarized in Table 1.

### 3.1. Growth Curve Models

Depression and anxiety were investigated separately. For both depression and anxiety, we conducted a random intercept-only model (the most basic form of growth model), followed by unconditional linear and quadratic growth curve models. For both depression and anxiety, the AIC and BIC indicated linear models fit the data best (Table 2). Estimated unconditional model means and conditional model means are plotted in Figure 1.

#### 3.1.1. Unconditional Depression Model

Results of the linear unconditional models for depression (Table 3) indicated that, for initial depression scores, both the intercept and slope factor were significantly different from zero (MINTERCEPT = 4.785, *p* < 0.01; VarINTERCEPT = 7.794, *p* < 0.01). Depression scores remained relatively stable over time (MLINEAR = 0.082, *p* = 0.516). For the slope factor, there was a significant amount of individual differences in the slope values around the mean growth curve (VarLINEAR = 0.963, *p* < 0.05), suggesting parents differed in their initial depression scores and their trajectories. In our model, the intercept and slope factors were negatively correlated (*r* = −0.382, *p* < 0.05) suggesting parents with higher initial depression scores tended to show smaller increases. Furthermore, the R2 values showed that between 46% and 68% of observed individual differences were accounted for by the growth factors. Regression coefficients and standard errors for depression are shown in Table 3.

#### 3.1.2. Conditional Depression Model

Results of the linear conditional models for depression indicated that, for initial depression scores, the intercept was significantly different from zero (MINTERCEPT = 13.997, *p* < 0.01) and there were significant individual differences in the slope values around the mean growth curve (VarLINEAR = 1.142, *p* < 0.01), suggesting parents differed in their initial depression scores and their trajectories. In the conditional model, the intercept and slope factors were not significantly correlated (*r* = −0.262, *p* = 0.252). When adjusting for predictor variables in the conditional model, depression scores decreased (MLINEAR = −3.600, *p* < 0.05). Higher W1 child internalizing scores (*r* = 0.401, *p* < 0.01) and lower parental sense of competency (*r* = −0.546, *p* < 0.001) were associated with higher initial levels of parental depression symptoms. For the slope, higher levels of parental anxiety and sense of competency at W1, as well as adopting an older child were associated with a steeper decrease of depression across time (*r* = 0.243, *p* < 0.05, *r* = 0.347, *p* < 0.01 and *r* = 0.439, *p* < 0.01, respectively). The R2 values showed that between 53% and 64% of observed individual differences in the conditional model were accounted for by the growth factors. Regression coefficients and standard errors for depression are shown in Table 3.

#### 3.1.3. Unconditional Anxiety Model

Results of the linear unconditional models for anxiety were similar to depression. The intercept and slope factor were significantly different from zero (MINTERCEPT = 5.250, *p* < 0.01; VarINTERCEPT = 3.228, *p* = 0.164). On average, anxiety scores remained stable (MLINEAR = 0.639, *p* < 0.01). For the slope factor, there were non-significant individual differences in the slope values around the mean growth curve (VarLINEAR = −0.569, *p* = 0.103), suggesting parents differed in their initial anxiety scores only. In our model, the intercept and slope factors were not significantly correlated (*r* = −0.331, *p* = 0.075). Furthermore, the R^2^ values indicated that between 29% and 59% of observed individual differences were accounted for by the growth factors. Regression coefficients and standard errors for anxiety are shown in Table 3.

#### 3.1.4. Conditional Anxiety Model

Results of the linear conditional models for anxiety showed that both the intercept and slope factor were significantly different from zero (MINTERCEPT = 14.675, *p* < 0.01; VarINTERCEPT = 6.986, *p* < 0.01). Results showed that anxiety symptom scores remained stable across time (MLINEAR = 0.604, *p* = 0.793). The conditional model showed a significant amount of individual differences in the slope values around the mean growth curve (VarLINEAR = 0.994, *p* < 0.05). The intercept and slope factors were not significantly correlated, (*r* = −0.076, *p* = 0.469). The only factor that predicted higher initial anxiety levels was a lower parental sense of competency (*r* = −0.383, *p* < 0.05). The R^2^ values indicated that between 71% and 90% of observed individual differences were accounted for by the growth factors. Regression coefficients and standard errors for anxiety are shown in Table 3.

## 4. Discussions

We investigated the longitudinal course of risk factors associated with depression and anxiety symptoms in parents who adopted children exclusively from the UK public care system at four time points across a four-year period. We used well-validated, standardized measures with parents of children adopted from foster care, in order to expand upon previous research and contribute to clinical and social work practice. Although adoptive parents’ reports of their mental health did not reveal depression prevalence rates as high as some studies of US adoptive mothers [4], in the present study, parents’ scores indicated higher rates of clinical symptoms of depression (4–12%) and anxiety (10–17%) compared to the general population using the same measure (4% depression, 13% anxiety; [58]). As such, this study underscores the need to support parental mental health over the early years of parenting, for example, [59], and for this to include adoptive parents. 

We charted patterns of change in adoptive parents’ symptoms of anxiety and depression as a function of their initial depression and anxiety scores, their sense of competency, and of child characteristics. When trajectories of parents’ depression were modelled as a function of the covariates, parents’ symptoms of depression reduced. Children’s internalizing symptoms five months post-placement were associated with higher initial symptoms of depression, suggesting that clinically-led training for adoption support teams to recognize and enable parents to cope with symptoms of depression and anxiety in children might hold some benefits for the manner in which families are supported through the initial stages of an adoptive placement. More generally, there is a clear need to acknowledge the increased caregiver stress and physical strain (e.g., exhaustion) associated with caring for a child who has experienced more pre-placement early adversity [7,30] and the potential for a contagion of negative affect associated with dysphoria within the family system [60].

In line with our hypotheses, parents’ sense of competence (their perception of their skills, or, self-efficacy and satisfaction with the role of being a parent) was associated with lower initial levels of anxiety and depression. A higher sense of competency was associated with a steeper decline in depressive symptoms over four years post-placement. Parents who report a lower sense of competency experience increased levels of parenting-related stress and emotional arousal in challenging parenting situations; they may be less able to put parenting knowledge into action and show less persistence in parenting tasks [36]. Additionally, parents who feel less in control of their children’s behavior are more likely to use negative parenting strategies [61]. As parents gain experience raising children, their self-efficacy and sense of satisfaction usually increase, but the persistence of difficult behaviors can impact upon parents’ assessments of their own abilities [62]. Efforts to support adoptive parents’ feelings of self-competence may be one pathway through which the intergenerational consequences of poor mental health and family crisis could be prevented.

Unexpectedly, children being older at the time of placement and higher parent ratings of their own symptoms of anxiety were associated with a steeper decline in depressive symptoms over four years post-placement. Possibly, adopting a school-aged child may provide parents with a routine and wider network of parents and teachers that provides additional sources of emotional and practical support and links to child-rearing advice that buffers parents’ feelings of being overwhelmed [63]. It is also possible that parents who rate themselves as more anxious in the early stages of a placement employ adaptive strategies (e.g., planning, positive reappraisal) that increase emotion regulation, coping, and resilience [64,65]. However, both these speculations warrant further study.

### Limitations and Future Directions

Some limitations of the present study are noteworthy. Parents were invited to take part once a child had been placed with them through the local authority, meaning that baseline measures of parent depression and anxiety were not obtained until approximately five months after the children were placed in their adoptive homes. The practical challenges of including psychological assessments pre-placement are significant; however, overcoming these would address mixed evidence regarding rates of prevalence pre- and post-adoption [10,13] and enable researchers to account for parents’ pre-adoptive experiences such as coping with infertility [66] and/or previous ‘matches’ with children that do not proceed to placement with concomitant periods of recovery from stress, grief and loss, including depressive symptoms [67]. Longitudinal assessment pre- and post-placement would also enable explicit investigation of Foli’s theory of parental post-adoption depression [39] in the context of UK adoption, as dissonance between parental expectations pre-placement (of self as parent, of the child, of family and friends, and of society) and the lived experience of family life may provide some explanation for parents’ symptoms of depression and anxiety.

The prevalence of clinically relevant symptoms of anxiety and depression reported in the present study was somewhat lower than other studies of adoptive families in other contexts (e.g., in different countries and within different processes of adoption, e.g., [4,13]). These may be, at least in part, attributable to the financial benefits, entitlement to paid leave available for maternity, and support services available to adoptive families residing in the UK [68,69]. Although future studies would do well to investigate the impact of pre- and post-adoption support available in different contexts of adoption, there are limitations in this study that may have led to an underestimation of prevalence rates in the sample. It is possible that disclosure for adoptive parents is challenging [10], given the feelings of guilt and shame associated with symptoms of depression, and that adopters tend to have high expectations of themselves [39]. Additionally, the self-report measures to assess depression and anxiety symptoms, parental sense of competency, and child behavior―reflecting cost and time constraints―may produce shared method variance that confounds the pattern of associations noted between these factors [70]. Future work should include other measures of adoptive parents’ mental health, such as interviews, or overcome the subjectivity of self-reporting entirely by using data linkage designs to access hospital and general practitioner records [71,72]. Finally, consistent with the restriction of range in the environment that is common of adoptive families [73], parents in our sample were generally well-educated with high incomes.

## 5. Conclusions

This study draws attention to the potential post-adoption support needs of parents and underscores the need to evaluate the efficacy of models of delivery via statutory and third sector support services, for example, support groups and mentoring [7]. The paucity of information about the relative value of such programmes and services needs to be addressed so that these scant resources can be deployed effectively to help parents (for an exception see [74]). Our findings add to the understanding of risk factors associated with depression and anxiety symptoms in adoptive parents, which could help general and more specialized practitioners be more attuned to the needs of adoptive families and to provide appropriate support and interventions. Increasing adopters’ awareness and understanding of the challenges of adoptive family life may also reduce barriers and stigma associated with seeking support and empower parents to access available support more readily.

## Figures and Tables

**Table 1:** Means, standard deviations, and correlations of conditional model variables.

	*N*	Mean	SD	1.	2.	3.	4.	5.	6.	7.
1. W1 depression score	96	4.72	3.43	-						
2. W1 anxiety score	96	6.10	3.48	0.611 **	-					
3. Age child placed for adoption	84	2.32	2.23	0.254 *	0.252 *	-				
4. W1 child internalizing	58	5.28	3.35	0.340 *	0.209	0.315 *	-			
5. W1 child externalizing	58	8.33	3.97	0.321 *	0.153	0.338 *	0.513 **	-		
6. Sibling group adoption	84	0.27	0.45	0.398 **	0.309 **	0.513 **	0.205	0.188	-	
7. W1 sense of competency	94	74.11	12.62	−0.562 **	−0.531 **	−0.428 **	−0.223	−0.312 *	−0.364 **	-

*Note*: * *p* < 0.05, ** *p* < 0.01.

**Table 2:** Fit indices for growth curve models using maximum likelihood with robust standard errors (MLR) based on missing at random (MAR) assumptions (*N* = 96).

	χ^2^	*df*	RMSEA	TLI	CFI	AIC	BIC
**Depression**							
M1 intercept only	17.006 *	8	0.108	0.887	0.849	1574.821	1590.207
M2 Unconditional linear	1.436	5	0.000	1.072	1.000	**1564.875**	**1587.955**
M3 Unconditional quadratic	**0.754**	1	0.000	**1.025**	1.000	1572.164	1605.501
M4 Conditional linear	14.300	17	0.000	1.061	1.000	798.148	838.300
**Anxiety**							
M1 intercept only	22.167 *	8	0.136	0.905	0.874	1629.025	1644.411
M2 Unconditional linear	3.191	5	0.000	**1.019**	1.000	**1615.244**	**1638.323**
M3 Unconditional quadratic	**0.440**	1	0.000	1.030	1.000	1620.664	1654.000
M4 Conditional linear	13.347	17	0.000	1.063	1.000	837.734	877.887

*Note*: χ^2^ = Chi-square value; *df* = degrees of freedom, RMSEA = root mean square error of approximation; SRMR = standardized root mean square residual; TLI = Tucker Lewis Index; CFI = Comparative Fit Index (CFI). Values in bold represent best fitting indices.

**Table 3:** Parameter estimates for unconditional and conditional linear models using MLR based on MAR assumptions (*N* = 96).

	Depression		Anxiety	
	Unconditional Linear Model Parameter Estimate (SE)	Conditional Linear Model Parameter Estimate (SE)	Unconditional Linear Model Parameter Estimate (SE)	Conditional Linear Model Parameter Estimate (SE)
**Intercept on**				
W1 Anxiety score ^a^		0.223 (0.156)		
W1 Depression score ^a^				0.277 (0.161)
Age placed ^a^		−0.267 (0.146)		−0.219 (0.125)
W1 Internalizing ^a^		**0.401 (0.152)**		0.209 (0.190)
W1 Externalizing ^a^		0.004 (0.139)		−0.098 (0.197)
Sibling group ^a^		0.137 (0.132)		0.050 (0.125)
W1 Parental sense of competency ^a^		**−0.546 (0.144)**		**−0.383 (0.152**)
**Slope on**				
W1 Anxiety score ^a^		**0.243 (0.119)**		
W1 Depression score ^a^				−0.316 (0.234)
Age placed ^a^		**0.439 (0.137)**		0.002 (0.253)
W1 Internalizing ^a^		−0.235 (0.174)		−0.257 (0.289)
W1 Externalizing ^a^		−0.294 (0.164)		0.446 (0.271)
Sibling group ^a^		0.111 (0.164)		0.013 (0.212)
W1 Parental sense of competency ^a^		**0.347 (0.164)**		−0.054 (0.256)
**Means/intercept**				
Intercept	**4.785 (0.337)**	**13.997 (3.774)**	**5.250 (0.318)**	**14.675 (4.527**)
Slope	0.082 (0.126)	**−3.600 (1.515)**	0.639 (0.126)	0.604 (2.299)
**Variances**				
Intercept	**7.794 (2.134)**	2.451 (2.135)	3.228 (2.318)	**6.986 (2.480)**
Slope	**0.963 (0.378)**	**1.142 (0.426)**	−0.569 (0.349)	**0.994 (0.438)**
**Intercept-slope correlation** ^a^	**−0.382 (0.149)**	−0.262 (0.228)	−0.331 (0.225)	−0.076 (0.339)

Note: SE =standard error. ^a^ The parameter estimate pertaining to these variables are standardized regression weights. Parameter estimates that are bolded are significant at *p* < 0.05.
